# Supplementary material for: Proximal immune-epithelial progenitor interactions drive chronic tissue sequelae post COVID-19
Source: Res Sq. 2023 Nov 28:rs.3.rs-3587418. Preprint. [Version 1] doi: 10.21203/rs.3.rs-3587418/v1 (PMC10705705; doi:10.21203/rs.3.rs-3587418/v1)
Supplement: Supplement 1 [file NIHPPrs3587418v1-supplement-1.pdf]

Supplementary Table1. Clinical information of the PASC-PF cohort.

Patient #	Duration of hospitalization during acute COVID-19 (Days)	Number of COVID-19 infections	Home O_2 supplement	Days from COVID-19 infection to lung transplant (Days)	CT Chest findings (pre-transplant)
1	30	4	Yes	109	1. Moderate left loculated hemothorax and bilateral pneumothoraxes.<br>2. Residual extensive bilateral COVID pneumonia/ARDS.
2	4	1	Yes	566	1. Persistent bronchiectasis, volume, and architectural distortion in upper lobes.<br>2. Stable bi-apical cystic structures with communicating bronchi.<br>3. Loculated left pneumothorax and bilateral pleural effusion
3	20	1	No	434	1. Diffuse areas of central bronchiectasis extending to the peripheries of both lungs with diffuse ground glass opacification.<br>2. Upper lobe predominant curvilinear areas of opacification suggesting fibrosis and scarring.<br>3. A 5 mm right upper lobe calcified granuloma.
4	90	1	Yes	456	1. Cystic bronchiectasis in the right upper lobe on a background of diffuse ground glass opacification, which may be sequela of COVID-19, though not typical.<br>2. Probable recurrent infection in the right upper lobe accounts for this appearance. Distribution is inconsistent with UIP.
5	149	1	Hospitalized until lung transplant	149	1. Moderate-sized bilateral apical pneumothoraxes.<br>2. Diffuse ground-glass opacification and bronchial retraction with subpleural blebs in this patient with known COVID-19 pneumonia and clinically confirmed ARDS, likely organizing/exudative phase and with possible diffuse alveolar damage.<br>3. Trace bilateral pleural effusions.<br>4. Mildly enlarged multi compartmental mediastinal lymph nodes are likely reactive.
6	Unknown	1	Yes	230	1. Small bleb in the left upper lobe.<br>2. Extensive interstitial ground-glass opacities as well as curvilinear consolidation likely related to sequelae from known Covid pneumonia.
7	Unknown	1	Yes	148	1. There is evidence for pulmonary fibrosis with traction bronchiectasis and honeycombing

					there is involvement of upper mid and lower lung zones.<br>2. Some increasing opacity and ground-glass of the bases may be progressive disease versus some superimposed active alveolitis.<br>3. There is mild mediastinal and hilar lymphadenopathy.
8	90	2	Yes	902	1. Chronic interstitial lung diseases.<br>2. Bilateral bronchiectasis greater on the right.<br>3. Reticular ground glass opacities in both lungs.
9	6	1	Yes	817	1. Diffuse bilateral pulmonary interstitial thickening, and bronchiectasis, most pronounced at the right lower lobe.
10	Unknown	2	Yes	150	1. Bilateral moderate to severe bronchiectasis centrally with areas of diffuse and severe peribronchial wall thickening especially in the lower lobes with several areas of mucous plugging.<br>2. Minimal areas of ground glass patchy airspace opacities are present within the left upper lobe and superior segment of the right lower lobe.
11	Unknown	1	Yes	300	1. Peripheral and peri-bronchovascular reticulations and textured ground-glass opacities without honeycombing, with associated architectural and pleural-parenchymal interface distortion, traction bronchiectasis and bronchiectasis, and regional volume loss, compatible with pulmonary fibrosis.
12	Unknown	1	Yes	53	1. A couple of < 5 mm right middle lobe calcified granulomas.<br>2. Extensive ILD likely sequelae of C19 pneumonia with diffuse ground-glass throughout the lungs.
13	38	2	Intubated	92	1. Dense consolidation with wedge-shaped areas of scarring are present bilaterally. Dense air bronchograms are present.<br>2. There is an air-containing cysts seen within the right middle lobe.<br>3. There is a specific mucus seen within the trachea and right mainstem bronchus.
14	60	1	Yes	80	1. Consolidations within the upper anterior segments bilaterally, w/ associated cystic traction bronchiectasis.<br>2. Diffuse consolidative and ground glass opacities throughout the remainder of the lungs.

					3. Large left pneumothorax with multiple adhesions along the anterior left upper lobe.<br>4. Small left pleural effusion.<br>5. Small right hydropneumothorax.
15	201	1	Hospitalized until lung transplant	201	1. Severe dense consolidation of both lungs with air bronchograms.<br>2. The right lung is collapsed with a right hydropneumothorax present.<br>3. Small left pleural effusion
16	58	2	Yes	120	1. Severe diffuse parenchymal lung consolidation with severe volume loss.<br>2. Moderate size loculated left pleural effusion and small loculated right pleural effusion.
17	20	1	Yes	329	1. Evidence of interstitial fibrosis manifested by predominantly subpleural upper zone reticulations and traction bronchiectasis with scattered areas of geographic perfusion and ground-glass opacities. This is all most likely residual of the patient's known Covid pneumonia.
18	0	1	Yes	748	1. Extensive changes of pulmonary fibrosis in the right greater than left lungs characterized by bibasilar predominant bronchiectasis, reticulation and honeycombing in near total involvement of the right lung with some relative sparing at the right lung apex. Extensive involvement of the left lower lobe and lingula with the left upper lobe slightly less involved.

Supplementary Table 2. Clinical Information of COVID-19 Convalescent Subjects.

	**Normal PFT**[1] N = 29	**Abnormal PFT**[1] N = 39	**p-value**[2]
**Age (years)**	51 (15)	50 (12)	0.12
**Sex**			0.80
Female<br>Male	13 (45%)<br>16 (55%)	16 (41%)<br>23 (59%)	
**Race**			0.60
White<br>Black<br>Other	15 (52%)<br>5 (17%)<br>9 (31%)	23 (59%)<br>8 (21%)<br>8 (21%)	
**Ethnicity**			0.60
Hispanic<br>Non-Hispanic	8 (28%)<br>21 (72%)	13 (33%)<br>26 (67%)	
**Admitted to Hospital**	22 (76%)	38 (97%)	0.009
Duration (Days)	14 (16)	21 (25)	0.14
**Time since Illness (Days)**[3]	113 (110)	98 (104)	0.80

[1]Median (SD); n (%)

[2]Wilcoxon rank sum test; Pearson's Chi-squared test; Fisher's exact test

[3]Earliest date: First symptoms, positive test, hospital admission

Supplementary Table3. Key resources.

**Reagent or resource**	**Source**	**Identifier**
**Antibodies (Immunofluorescence)**		
Rat anti-cytokeratin 8 (TROMA-1)	DSHB	Antibody Registry ID: AB 531826
Rabbit anti-proSP-C	Millipore Sigma	Cat#: AB3786
Hamster anti-PDPN	Abcam	Cat#: ab11936
Chicken anti-cytokeratin 5 (human)	BioLegend	Cat#: 905901
Chicken anti-cytokeratin 5 (mouse)	BioLegend	Cat#: 905903
Rabbit anti-CD8α (human)	Cell Signaling Technologies	Cat#: 85336S
Rabbit anti-CD8α (mouse)	Cell Signaling Technologies	Cat#: 98941S
Rabbit anti-cytokeratin 17	Millipore Sigma	Cat#: HPA000453
Mouse anti-SMA	Millipore Sigma	Cat#: A5528
Rabbit anti-AGER	Proteintech	Cat#: 16346-1-AP

Mouse anti-CD68	Abcam	Cat#: Ab955
Mouse anti-proSP-C	Santa Cruz	Cat#: sc-518029
Rabbit anti-CX3CR1	ThermoFisher	Cat#: 14-6093-81
Mouse anti-TNF	Abcam	Cat#: ab1793
Rabbit anti-IFNγ	Proteintech	Cat#: 15365-1-AP
DyLight 649 Donkey anti-rabbit IgG	BioLegend	Cat#: 406406
Donkey anti-chicken IgGY (H+L) Alexa Fluor 488 IgG (H+L)	ThermoFisher	Cat#: A78948
Goat anti-rat IgG (H+L) Alexa Fluor 555	ThermoFisher	Cat#: A48270
Goat anti-Armenian Hamster IgG (H+L) Alexa Fluor 488	ThermoFisher	Cat#: A78963
Goat anti-Chicken IgY (H&L) Alexa Fluor 750	Abcam	Cat#: ab175755
Goat anti-rat IgG (H+L) Alexa Fluor 488	ThermoFisher	Cat#: A-11006
Goat anti-rabbit IgG (H+L) Alexa Fluor 488	ThermoFisher	Cat#: A-11008
**Antibodies (Flow cytometry)**		
Viability dye (Zombie NIR)	BioLegend	Cat#: 423105
Viability dye (Zombie Aqua)	BioLegend	Cat#: 423101
CD8a-BV510 (53-6.7)	BioLegend	Cat#: 100751
CD11b-BV510 (M1/70)	BioLegend	Cat#: 101245
CD11b-PerCP/Cy5.5 (M1/70)	BioLegend	Cat#: 101227
CD11c-BV421 (N418)	BioLegend	Cat#: 117329
CD45-BV605 (30-F11)	BioLegend	Cat#: 103139
CD64-BV711 (X54-5/7.1)	BioLegend	Cat#: 139311
IA/IE–PerCP/Cy5.5 (M5/114.15.2)	BD Biosciences	Cat#: 556999
IL-1b-PE (NJTEN3)	eBioscience	Cat#: 12-7114-82
Ly6C-PE (HK1.4)	BioLegend	Cat#: 128007
Ly6G-PE/Cy7 (1A8)	BioLegend	Cat#: 127617
MerTK-APC (2B10C42)	BioLegend	Cat#: 151507
Influenza $NP_{366-374}$-APC	NIH	IEDB ID: 4602

SiglecF-APC/Cy7 (E50-2440)	BD Biosceinces	Cat#: 155531
CD45-APC (30-F11)	BioLegend	Cat#: 103111
EpCAM/CD326 – PerCP/Cy5.5 (G8.8)	BioLegend	Cat#: 118219
IA/IE-FITC (M5/114.15.2)	BioLegend	Cat#: 107605
**Chemicals, Peptides, and Proteins**		
Dispase II (Neutral protease, grade II)	Roche	Cat#: 4942078001
DNase I	Sigma-Aldrich	Cat#: D4527-10KU
Murine IL-1β	Peprotech	Cat#: 211-11B
**Primers**	**Forward**	**Reverse**
Vegfa	CCGGTTTAAATCCTGGAGCG	TTTAACTCAAGCTGCCTCGC
Clic5	ATGACGGACTCAGCGACAAC	GTAGATCGGCTGGCTTTCTTTT
Aqp5	TCTTGTGGGGATCTACTTCACC	TGAGAGGGGCTGAACCGAT
Hif1a	ACCTTCATCGGAAACTCCAAAG	ACTGTTAGGCTCAGGTGAACT
Cldn4	GTCCTGGGAATCTCCTTGGC	TCTGTGCCGTGACGATGTTG
Slc16a3	TCACGGGTTTCTCCTACGC	GCCAAAGCGGTTCACACAC
Slc2a1	CAGTTCGGCTATAACACTGGTG	GCCCCCGACAGAGAAGATG
Il1b	GCAACTGTTCCTGAACTCAACT	ATCTTTTGGGGTCCGTCAACT

Supplementary Table4. Spatial transcriptomics gene sets

**Cell type/Signaling pathway**	**Marker genes/source**
**MOUSE**	
CD8 T cells	*CD8a, CD8b1, Itgae, Cd3d, Trbc1, Ccl5, Gzma, Gzmb*
Monocyte derived macrophages	*Cd14, S100a6, Apoe, Mafb, Vcan, Fn1, Stat1*
Alveolar macrophages	*Sparc, Flt1, Fabp5, Car4, Krt79*
Alveolar epithelium	*Sftpc, Clic5, Emp2, Hopx, Spock2, Sftpa1, Cldn18*
Krt-rich dysplastic repair	*Krt5, Krt8, Krt17, Trp53, Cldn4*
ADI/PATS/DATP signature	(20), (21), (22)
Aberrant basaloid signature	(23), (24)

Fibrosis	WP_Lung_Fibrosis
IL-1R signaling	BIOCARTA_IL1R_PATHWAY (GSEA; Mouse)
Inflammasome signature	REACTOME_INFLAMMASOMES (GSEA; Mouse)
**HUMAN**	
CD8 T cells	CD8A, CCL5, GZMH, GZMA, GZMK, GZMB, GNLY
Monocyte derived macrophages	CD14, RNASE1, S100A8, SPP1, TYMP, MS4A6A, FCGR2B
Alveolar macrophages	GPD1, INHBA, MME, APOC1, CD52, PCOLCE2, PPARG, FABP4, SCD
Alveolar epithelium	AGER, CLDN18, ABCA3, LAMP3, SFTPA1, SFTPD, SFTPC, CLIC5, AQP4
Krt-rich dysplastic repair	KRT8, KRT5, KRT17, MMP7, ITGB6, AQP3, KRT19, MMP1, S100A2
IL-1R signaling	BIOCARTA_IL1R_PATHWAY (GSEA; Human)
Inflammasome signature	REACTOME_INFLAMMASOMES (GSEA; Human)
IFN + TNF signaling	REACTOME_INTERFERON_GAMMA_SIGNALLING (GSEA Human), HALLMARK_TNFA_SIGNALING_VIA NFKB (GSEA; Human)
